# Caring for Psychological Distress of Patients With COVID-19: A Mixed-Method Cross-Sectional Study

**DOI:** 10.3389/fpsyg.2022.766036

**Published:** 2022-04-06

**Authors:** Juan Li, Anni Wang, Lei Liu, Xue Chen, Xiaoling Bai

**Affiliations:** ^1^Xiangya School of Nursing, Central South University, Changsha, China; ^2^Department of Nephrology, The First Affiliated Hospital, Guizhou University of Traditional Chinese Medicine, Guiyang, China; ^3^Department of Nursing, Chinese Medicine Hospital of Longli County, Longli, China; ^4^Guizhou Nursing Vocational College, Guiyang, China

**Keywords:** COVID-19, patients, psychological distress, healthcare workers, mixed-method

## Abstract

**Introduction:**

The 2019–2020 pandemic COVID-19 has become a global health crisis. While many recent studies on COVID-19 pandemic have focused on disease epidemiology and psychological status of patients, few have explored the multi-facet influential factors or combined perspectives from both the patients and healthcare workers. The purposes of this study were to: analyze the influencing factors of psychological distress of COVID-19 patients; and describe the experience of healthcare workers relieving psychological distress.

**Materials and Methods:**

This study uses a mixed-method cross-sectional design, including a quantitative study and a qualitative study, targeting two populations: COVID-19 patient and health workers, respectively. In the quantitative part, we recruited a convenience sample of patients with COVID-19 from five hospitals in Wuhan, Hubei Province from 10 to 15 April, 2020. Besides, we collected data by using participants’ socio-demographic information sheet, the Connor-Davidson Resilience Scale-10, the Herth Hope Index, the Distress Thermometer, the Revised Chinese Version of Mishel Uncertainty in Illness Scale, and the Chinese Version of Wake Forest Physician Trust Scale. In the qualitative part, the participants were healthcare workers involved in medical aid missions in Hubei Province, China. Meanwhile, we used sampling with convenient and purposive, data collection with a semi-structured online video interview, and text transcription with Colaizzi’s phenomenological method.

**Results:**

The results reveal that 25.7% of patients reported higher level of psychological distress (*n* = 31, scoring ≥4). After controlling the sociodemographic variables, only severity of COVID-19 (β = 0.282, *P* = 0.025) and uncertainty in illness (β = 0.345, *P* = 0.007) shown significant effect on psychological distress in the regression model (*F* = 10.862, *R*^2^ = 0.222, *P* < 0.001). The experience of healthcare workers emerged five themes: Particularly needed psychological care, Manifestation of negative emotion, Manifestation of proactive adaptation, Strategies relieving psychological distress, and gains of healthcare workers after delivering effective psychological care.

**Conclusion:**

The 25.7% of patients with COVID-19 still suffered from psychological distress, which should receive timely attention from healthcare workers. And the severity of the disease and disease uncertainty have a significant impact on distress. It is critical to train the healthcare workers on detecting the different manifestation of psychological distress, offering timely disease related information, and applying communication strategies.

## Introduction

The coronavirus disease 2019 (COVID-19) epidemic in December 2019 was regarded by the World Health Organization as a “public health emergency of international concern” (Word Health Organization, 2020). Currently, with the strengthening of protective measures and the popularization of global vaccination, the situation of COVID-19 in some areas of the world has been basically controlled, but there are still some areas in the prevention and control dilemma (Khan et al., 2020; Halder et al., 2021). On the one hand, confirmed patients have to face unknown results or even death threats caused by the coronavirus infection. On the other hand, they have to face the influence of factors such as the unfamiliar environment because of isolation treatment, the well protected medical staff, the spread of untrue information, and so on, which poses a challenge to psychological condition in most patients. Previous studies have shown that more than 50% of patients have suffered from anxiety and fear during the epidemic. Zhang et al. (2020) revealed that 20.9% of patients with mild symptoms of COVID-19 have anxiety disorders and 18.6% of patients have depression disorders. Bo et al. (2021) also found that 96.2% of confirmed patients of Fangcang hospitals experienced post-traumatic stress disorder.

Psychological distress refers to an unpleasant emotional experience experienced when coping with stress caused by multiple factors (Ridner, 2004; Barlow and Durand, 2011). It is essentially psychological (cognitive, behavioral, and emotional), social, and spiritual changes. Psychological distress was initially regarded as an emerging variable in the field of cancer care, and was considered as one of the signs of life cancer care. The incidence of psychological distress in cancer patients was 24.2–37.8%. It is also widely used in the healthcare areas such as diabetes and schizophrenia, etc. (Indelicato et al., 2017; Wang A. N. et al., 2020). Studies have shown that psychological distress can show normal emotional reactions such as fragility, sadness, and fear, as well as serious manifestations, such as depression, anxiety, fear, social isolation, existence, and mental crisis. Many large-scale studies have confirmed that, it is very common for patients to experience psychological distress and will bring many negative effects, such as affecting the quality of life, pain, adherence to treatment and satisfaction, etc. Considering the prevalence of psychological distress among patients and its negative impact on the quality of life, routine screening of patients’ psychological distress is recommended internationally as one of the necessary items of medical care. At the same time, for the target group of patients experiencing outbreaks of infectious diseases, psychological distress is easier to understand than anxiety and depression, and its clinical manifestations are more comprehensive. Therefore, it is necessary to carry out psychological distress screening among COVID-19 patients.

Existing evidence has focused on the influencing factors of cancer patients’ psychological distress, but the influencing factors obtained by various studies are not consistent, and each study has not included multiple influencing factors at the same time (Di, 2015; Chen et al., 2020; Tian et al., 2021). However, few researchers discuss the psychological distress level of COVID-19 patients, let alone analysis of possible influencing factors, which is not conducive to the early identification of high-risk groups of COVID-19 patients with psychological distress, and it is impossible to formulate targeted intervention programs, which may affect the patient’s ability to cope with the coronavirus and the quality of life.

At present, few studies have revealed the condition of psychological distress of COVID-19 patients and its influencing factors. According to King’s theory of goal attainment (King, 1992), it can be known that in the health care system, nurses and patients form an interpersonal system, which illustrate that nurses are helpers with professional knowledge and skills, while patients are those who have nursing needs and caring problems. It can be seen that the psychological distress of patients with outbreaks of infectious diseases will be affected by intrapersonal factors and interpersonal factors (Shechter et al., 2020).

Through a comprehensive literature review, it can be found that intrapersonal factors may include socio-demographic information (age, gender, family residence, etc.) (Liu N. et al., 2020; Shahrour and Dardas, 2020), disease related information (disease type, treatment stage, informed disease information, etc.) (Li et al., 2020), resilience, and hope (Qiu et al., 2020). For example, firstly, research on coping strategies of cancer patients shows that men take more active coping and avoidance ways than women (Zhang et al., 2010); patients living in rural areas have a higher risk of psychological distress than those living in cities, which is related to differences in regional economic and cultural levels; and there is a higher incidence of psychological distress in patients with advanced cancer (Salmon et al., 2015). Additionally, hope refers to a dynamic inner power that can transcend the *status quo* and generate positive values for life (Herth, 1992). Wang C. et al. (2020) suggested that inner strength and hopefulness are critical for adaptation, because the outbreaks of infectious diseases often cause patients to feel fear and hopelessness during the isolation process. Besides, previous researches (Hildon et al., 2010; Xiao et al., 2021) have suggested that resilience can be seen as a defense mechanism that enables individuals to thrive in adversity. The level of individual resilience determines the ability to cope with the crisis and the outcome of adaptation. Individuals with high psychological resilience can overcome various adversities and tend to have an optimistic and positive attitude to adapt to the environment.

In addition to intrapersonal factors, many studies have found that people potentially have the interpersonal strength to successfully adapt to stress (Zauszniewski et al., 2015), which may include uncertainty about disease and trust in healthcare workers (HCWs). As an explosive infectious disease, COVID-19 was initially unclear in terms of disease-related information and treatment, which undoubtedly caused great physical and mental impact on patients. Among them, the sense of disease uncertainty is an important factor (Mishel, 1990; Haisfield-Wolfe et al., 2012), which is defined as a sense of loss of control over disease related events and one’s future. Studies have proved that a high level of disease uncertainty will reduce the patient’s mobility, lifestyle and treatment compliance, and ultimately have a significant impact on the patient outcomes. Additionally, patients’ trust in HCWs is defined as the subjective willingness of patients to accept the inferior position relative to the HCWs in the absence of the ability to supervise and control the behavior of the other party, and the expectation of the HCWs to take the favorable behavior to the patients. And, the sense of patients’ trust in HCWs is one of the important criteria to evaluate the health workers-patient relationship (Kim et al., 2018), which influence patients’ adherence to treatment plan, thus affecting the prognosis of the disease.

At the same time, HCWs are one of the people who have participated the most in the fight against COVID-19, and in fact, they have regular direct contact with COVID-19 patients from admission to discharge (Galehdar et al., 2020). Therefore, more than any other group of people in this epidemic, HCWs are more aware of the psychological distress of COVID-19 patients. There are currently some researches on HCWs caring for COVID-19 patients (Chen et al., 2020; Walton et al., 2020), but the existing evidence still shows that there is insufficient nursing experience, especially for psychological nursing, and there is a lack of scientific psychological nursing empirical evidence guidance (Inchausti et al., 2020). Therefore, it is also necessary to explore the experience of the care of COVID-19 patients with psychological problems from the perspective of HCWs, which can objectively reflect the psychological distress conditions and nursing needs of patients. Hence, the purpose of this study is to acquire a more comprehensive understanding of the intrapersonal and interpersonal factors associated with the currently psychological distress and explore the experience of caring for patients with COVID-19 by combining patients’ self-reported psychological problems with the work experience of HCWs.

## Materials and Methods

### General Study Design

The study employed a mixed-method convergent design, including a quantitative study and a qualitative study, targeting two populations: COVID-19 patients and HCWs, respectively. This type of research design provides a more comprehensive perspective for understanding a complex phenomenon because it uses these two different research methods to collect data and cross-analyze the data (in our case, COVID-19 patients and HCWs). For the current work, the data of the two sub-studies that compare the results will better understand the intrapersonal and interpersonal factors related to current psychological distress, and explore the experience of caring for COVID-19 patients.

### Quantitative Survey of Patients

#### Recruitment and Data Collection

We employed a cross-sectional study and recruited a convenience sample of patients with COVID-19 from five hospitals (including two designated hospital, one newly built hospital, and two makeshift hospitals) in Wuhan, Hubei Province from 10 to 15 April, 2020.

Firstly, we obtained the ethics approval from the Institutional Review Board and the survey approval from nursing managers of the local hospital. Then, the recruitment notice for this study was circulated by two local nurses who worked as research assistants, through a WeChat group (a widely used social-media platform in China), and contact with potential participants was established. The inclusion criteria involved: (a) patients confirmed with coronavirus pneumonia according to the “Novel Coronavirus Pneumonia Diagnosis and Treatment Plan (Eighth Edition)” (National Health Commission of the People’s Republic of China, 2021), referring to having relevant clinical manifestations, such as fever, dry cough, fatigue, sore throat, loss of smell (taste), diarrhea, and other self-perceived or clinically recognizable symptoms and signs; have a history of exposure to patients with new coronavirus infection and asymptomatic infections within 14 days of onset; the coronavirus nucleic acid tested positive; (b) those who participated in the study voluntarily, with informed consent; and (c) those able to express their thoughts with clarity and consciousness. Correspondingly, the exclusion criteria involved: (a) people with previous severe mental or cognitive impairments, comprehension, memory, orientation, and other audio-visual impairments; and (b) those who participated in other related researches at the same time.

According to the Kendal’s sample size calculation principle that the sample size is 5–10 times the number of independent variables (Lewis, 2002). There were 15 variables in this study, including six socio-demographic factors, five disease related factors, two intrapersonal factors, and two interpersonal factors. Thus, the sample size was 90–180 with a 20% invalid response rate. Finally, a total of 120–130 patients with COVID-19 participated in the study (response rate: 92.3%).

#### Study Instruments

##### Self–Reported Sociodemographic and Clinical Information Sheet

The sociodemographic and clinical characteristics of participants were collected. These included participants’ age, gender, marital status, educational level, place of residence, average monthly earnings, duration of diagnosis, duration of hospitalization, complication, severity, and whether family members confirmed with COVID-19.

##### Distress Thermometer

Distress Thermometer (DT) is a visual analog scale and includes 11 numbers from 0 to 10 to represent the degree of psychological distress, where 0 means no distress, 1–3 points for mild distress, 4–6 points for moderate distress, 7–9 points for severe distress, and 10 points for extremely distress (Haverman et al., 2013), and the diagnostic threshold of the Chinese version is ≥4 points. The test-retest reliability of the Chinese version is 0.80, showing good reliability and validity (Tang et al., 2011).

##### The Connor-Davidson Resilience Scale-10

The Connor-Davidson Resilience Scale (CD-RISC) was first compiled by American psychologists Connor and Davidson (2003). It includes a 25-item self-assessment questionnaire to assess the level of individual psychological resilience. After further analysis by Campbell-Sills and Stenin, 10 items were extracted from 25 items, which constituted the CD-RISC single-dimensional scale. The scale uses 0–4 scores. The total psychological resilience score is added for each item (the score ranges from 0 to 40 points). The higher the score, the higher the individual’s psychological resilience level. The 10-item CD-RISC Chinese version was translated and revised by Wang et al. (2010), providing that the scale has good internal consistency coefficient (α = 0.91) and test-retest reliability (2 weeks interval *r* = 0.90), which is suitable for application in Chinese population.

##### The Herth Hope Index

The Herth Hope Index (HHI) is a tool to measure the level of hope (Herth, 1992). The scale contains 12 items, from 1 to 4 in three dimensions: temporality and future, positive readiness and expectancy, and interconnectedness. The higher the total score, the higher the level of hope. The Cronbach’s α coefficient of the Chinese version of HHI is 0.85 (Wang, 2000).

##### The Revised Chinese Version of Mishel Uncertainty in Illness Scale

Mishel Uncertainty in Illness Scale (MUIS) was compiled by Mishel in 1981, the revised Chinese version of MUIS was revised by Ye et al. (2018). The Chinese version of MUIS includes 22 items in three dimensions: vagueness, lack of clarification, and unpredictability, using Likert 5-level score. Each item is scored from 1 (strongly disagree) to 5 (strongly agree), with a total score of 22–110 points. The higher the score, the higher the patient’s disease uncertainty. The Cronbach’s α coefficient is 0.825, and the test-retest reliability coefficient is 0.836.

##### The Chinese Version of Wake Forest Physician Trust Scale

The Chinese Version of Wake Forest Physician Trust Scale (WFPTS) (Dong and Bao, 2012) has 11 items, divided into two dimensions: benevolence (representing the doctor’s caring attitude, communicating with patients, etc.) and technical ability (representing the content reflecting the doctor’s medical skill level). Each item is scored from 1 (strongly disagree) to 5 (strongly agree). The higher the total score represents the higher the patient has trust in the physician. The scale has good reliability and validity. The test-retest reliability of the total scale is 0.83, and the test-retest reliability is 0.83.

#### Data Analysis

Data were inputted and analyzed by using IBM SPSS 20.0 (SPSS Inc., Chicago, IL, United States). The count data was represented by relative number, χ^2^ test or Fisher’s exact probability method was used for intergroup comparison; the measurement data was expressed in terms of mean ($\bar{\text{X}}$) and standard deviation (SD), and *t*-test was used for intergroup comparison. We used hierarchical multiple regression analysis to analyse the influencing factors. A two–tailed *p* value < 0.05 was considered statistically significant.

### Qualitative Interviews With Healthcare Workers

#### Recruitment and Data Collection

We designed a descriptive phenomenological study, aimed at describing and exploring the meanings of respondents’ lived experiences. The participants were HCWs involved in medical aid missions in Hubei Province, China, where the initial outbreak of COVID-19 occurred. The participants were selected through convenience and purposive sampling.

The main researchers (XB, LL, and XC), female, nurses, joined the medical aid mission team and made the study known to HCWs by issuing electronic invitations post via the WeChat platform. Then, potential participants who agree to join the study can contact the research team by scanning the QR code displayed in the invitation. Totally, we contacted both integrated and mixed aid mission teams from five hospitals, and ended the e-invitation when data saturation. Furthermore, before the study began, the researchers and the participants did not know each other. After we further introduced the content of the study to participants, a total of five HCWs declined further interviews, mainly due to lack of interest or lack of time. After the 25th participant completed the interview, the data reached saturation and no new information was generated.

Since face-to-face interviews pose a risk of infection to both interviewers and interviewees, we managed to collect data through semi-structured in-depth video interviews conducted from 10 to 30 April, 2020. Besides, we recorded all interviews electronically, and also collected the participants’ gender, age, education level, professional qualification, working tenure, original working department, specialist qualification, duration of aid, position in aid team, mission site, and team form (see Table 1). Researcher ensure confidentiality by using numbers instead of names (for example, nurses N1, N2, etc. and physician P1, P2, etc.) and deleting identifying information from the record. And a semi-structured format with open-ended questions was used, developed by the research team based on relevant literature and own understanding. Illustrative questions were: “please describe the experience of providing psychological care to patients with COVID-19”; “what was the difference between providing psychological care and working in the original department due to the epidemic”; “what problems did you experiencing in providing psychological ca 247 re to patients”; “what help have you received”; “what other help did you need”; “how did you feel overall.” Probing questions, such as “please elaborate on that,” were used to elicit more information.

**TABLE 1:** Demographic characteristic of the healthcare workers (HCWs) (*N* = 25).

Participant	Gender	Age (Year)	Education level	Professional qualification	Working tenure (Year)	Original department	Specialist qualification	Duration of aid (days)	Position in aid team	Mission site	Team form
Nurse 1	Female	32	Bachelor	Senior nurse	9	Nephrology	None	30	Nurse	Designated hospital	Integrated
Nurse 2	Female	36	Bachelor	Senior nurse	18	Department of Infection Control	ICU specialist	30	Head nurse in ward	Designated hospital	Integrated
Nurse 3	Female	35	Bachelor	Registered Nurse	11	Gynecology	None	30	Nurse	Designated hospital	Integrated
Nurse 4	Female	35	Bachelor	Senior nurse	10	Urology	None	30	Chief head nurse	Designated hospital	Integrated
Nurse 5	Female	26	Bachelor	Registered Nurse	5	ICU	ICU specialist	30	Nurse	Designated hospital	Integrated
Nurse 6	Female	27	Bachelor	Registered Nurse	5	Oncology	None	30	Nurse	Designated hospital	Integrated
Nurse 7	Female	27	Bachelor	Registered Nurse	4	Cardiology	None	31	Nurse	Designated hospital	Mixed
Nurse 8	Female	28	Bachelor	Senior nurse	7	Gastroenterology	None	31	Nurse	Designated hospital	Mixed
Nurse 9	Male	32	Bachelor	Registered Nurse	9	ICU	None	42	Nurse	Designated hospital	Mixed
Nurse 10	Female	30	Bachelor	Senior nurse	10	ICU	ICU specialist	35	Nurse	Newly built hospital	Mixed
Nurse 11	Female	33	Bachelor	Senior nurse	10	Respiratory department	None	53	Nurse	Designated hospital	Mixed
Nurse 12	Male	29	Bachelor	Registered Nurse	7	ICU	ICU specialist	46	Nurse	Newly built hospital	Mixed
Nurse 13	Female	33	College	Registered Nurse	11	ICU	ICU specialist	45	Charge nurse in shift	Makeshift hospital	Mixed
Nurse 14	Female	36	Bachelor	Senior nurse	16	Nursing Management Department	None	44	Chief head nurse	Makeshift hospital	Mixed
Nurse 15	Female	28	College	Registered Nurse	4	ICU	ICU specialist	34	Nurse	Makeshift hospital	Mixed
Nurse 16	Male	30	Bachelor	Registered Nurse	5	Emergency department	None	52	Head nurse in ward	Designated hospital	Integrated
Nurse 17	Female	26	Bachelor	Registered Nurse	3	ICU	None	58	Nurse	Designated hospital	Integrated
Physician 18	Female	42	Master	Associate chief physician	17	Gastroenterology	None	30	Team leader	Designated hospital	Integrated
Physician 19	Male	32	Bachelor	Attending Physician	9	Respiratory department	None	30	Physician	Designated hospital	Integrated
Physician 20	Female	36	Master	Associate chief physician	10	Respiratory department	None	30	Physician	Designated hospital	Integrated
Physician 21	Female	41	Master	Associate chief physician	16	Endocrinology	None	30	Physician	Designated hospital	Integrated
Physician 22	Male	37	Master	Attending Physician	9	Nephrology	Peritoneal dialysis, hemodialysis	32	Physician	Newly built hospital	Mixed
Physician 23	Male	31	Master	Attending Physician	2	Rheumatology	None	36	Physician	Newly built hospital	Mixed
Physician 24	Male	42	Bachelor	Associate chief physician	17	Cardiology	None	27	Team leader	Newly built hospital	Mixed
Physician 25	Male	41	Bachelor	Associate chief physician	19	Nephrology	Peritoneal dialysis, hemodialysis	36	Team leader	Newly built hospital	Mixed

The interviews were conducted in Chinese by two researchers (XB and AW), with XB being the primary interviewer to ask questions and AW acting as an assistant to supplement questions and take notes. Both researchers are competent for qualitative research. In addition, XB, as a nurse, has worked in the hospital for nearly 10 years and has good communication skills with HCWs. Moreover, we conducted separate video interviews at a time convenient for participants, because the HCWs were quarantined after a shift at their designated dormitory room. Each interview was audio-recorded and lasted for 30–45 min.

In order to ensure the accuracy and reliability of the study, before the interview began, we sent the interview outline to other experts with extensive experience in qualitative research for review before the interview began. Additionally, after transcribing and forming the primary code, the researchers sent the text and code to participants in a WeChat message and asked them to determine whether the extracted code was consistent with the views they expressed. Moreover, we tried to purposefully increase the diversity of participants, such as gender, age, professional qualification, working tenure, duration of aid, etc.

#### Data Analysis

Data collection and data analysis are carried out at the same time. These recordings were transcribed verbatim within 24 h of the interview, and the accuracy was reviewed by the interviewer. The interviews, original transcripts, and data analysis were all written in Chinese. Researchers use Nvivo 10 software to use Colaizzi’s phenomenological method for data analysis and topic induction. Two researchers (AW and JL) double-coded the interview data to check consistency and accuracy. Then we discussed the coding differences until all researchers reached a consensus. After that, we reorganized and classified codes to generate different themes/sub-themes, and on this basis, we explored deeper issues in subsequent interviews. We stop data collection when the subject reaches saturation. AW was responsible for translating all citations into English, and JL was responsible for back translation to ensure that the meaning was preserved.

### Ethics Statement

Prior to collecting the data, the study was approved by the Institutional Review Board at the researchers’ institute (IRB number: 202025). Before filling out any questionnaire, all participants were informed of the purpose and procedures of the study, and verbal and written consent were signed which provided that the entire study was completely voluntary, anonymous, and conducted in secret. Furthermore, participants have the right to withdraw from the study at any time without impunity. The researchers conducted research when participants felt sufficiently energetic and well. Contact information for obtaining psychological assistance, if needed, was provided. All recordings and transcribed texts were anonymous and securely stored by the researchers.

## Results

### Quantitative Survey of Patients

#### Sociodemographic and Clinical Characteristics

In total, 120 participants were recruited for the study (response rate: 92.3%). As shown in Table 2, among all the patients, the mean age was 51.16 years (*SD* 15.39, range from 20 to 85), 57.5% of the patients are male, and the majority of patients lived in city (90.0%). Most patients were diagnosed for more than 21 days (83.3%), and 76.7% of the patients were hospitalized for more than 15 days. In terms of severity of disease, the patients with mild and common types accounted for the most, accounting for 73.4%, but only 26.6% of patients were severe and critical types.

**TABLE 2:** Sociodemographic and clinical characteristics of patients with mild symptoms of coronavirus disease 2019 (*N* = 120).

Variables	*N*	%
**Gender**		
Male	51	42.5
Female	69	57.5
**Marital status**		
Single	14	11.6
Married	89	74.2
Divorced	7	5.8
Spouse	10	8.4
**Education level**		
Primary school	34	28.3
Junior middle school	18	15.0
Senior middle school	34	28.3
Undergraduate	17	14.2
Postgraduate or above	17	14.2
**Residence**		
City	108	90.0
Town	7	5.8
Countryside	5	4.2
**Average monthly earnings (RMB)**		
≤2,000	24	20.0
2,001–4,000	45	37.5
4,001–6,000	24	20.0
≥6,000	27	22.5
**Duration of diagnosis (days)**		
≤7	3	2.5
8–14	12	10.0
15–21	5	4.2
≥22	100	83.3
**Duration of hospitalization (days)**		
≤5	10	8.3
6–10	12	10.0
11–15	6	5.0
≥16	92	76.7
**Complication**		
Yes	41	34.2
No	79	65.8
**Severity of COVID-19**		
Mild	47	39.2
Normal	41	34.2
Severe	26	21.6
Critical	6	5.0
**Family members confirmed with COVID-19**		
Yes	65	54.2
No	55	45.8

*We used convenience sampling to recruit patients with COVID-19 from five hospitals in Wuhan, Hubei Province, China.*

#### The Level of Distress and Its Influential Factors

The study shown that 25.7% of patients reported higher level of psychological distress (*n* = 31, scoring ≥4). In regression analysis, after controlling the sociodemographic variables, only severity of COVID-19 (β = 0.282, *P* = 2860.025) and uncertainty in illness (β = 0.345, *P* = 0.007) shown significant effect on psychological distress in the regression model (*F* = 8.937, *R*^2^ = 0.294, *P* = 0.07; see Table 3).

**TABLE 3:** Hierarchical multiple regression analysis of psychological distress among the patients with COVID-19.

Variables		Step1			Step2		
		β value	*T*	*P* value	β value	*T*	*P* value
Intrapersonal factors	Severity of COVID-19	0.371	2.966	0.004**	0.282	2.309	0.025*
Interpersonal factors	Sense of uncertainty				0.345	2.822	0.007**
*R* ^2^		0.138			0.294		
*F*		8.797			8.937		
*P*		0.004			0.007		

*P<0.05, **P<0.01.

### Qualitative Experience of Healthcare Workers in Psychological Care

A total of 25 HCWs, comprising 17 nurses and 8 physicians (17 females and 8 males), aged between 26 and 42 years (mean = 33 years; SD = 5), participated in the study. Their professional tenures ranged between 2 and 19 years (mean = 9.72; SD = 4.98). The duration of aid was 27–58 days (mean = 36.08; SD = 8.71).

By analyzing the work experience of HCWs in providing psychological care for COVID-19 patients, the researchers extracted five themes, namely “particularly needed psychological care,” manifestation of negative emotion,” “manifestation of proactive adaptation,” “strategies relieving psychological distress,” and “gains of healthcare workers after delivering effective psychological care,” respectively.

#### Particularly Needed Psychological Care

The first theme cluster identified was “particularly needed psychological care.” All participants agreed that the main differences between the work of caring for COVID-19 and previous work were that apart from caring for patients with infectious diseases in the isolation ward, another obvious problem was the psychological distress of the patients.

“The patients are quarantined and see the increasing number of infections every day. They are very worried and distressed and very dependent on you.” (N1)

When many HCWs just entered the setting, they did not pay much attention to it at the beginning. After going into the setting, they especially felt the emotional status of patients. Sometimes HCWs could clearly feel the anxiety of patients through some simple conversations during ward rounds and operations. “Patients are very eager to go to ask about their condition when they see physicians and nurses.” (N5)

From the experience of HCWs, the main reason for the patient’s significant psychological distress is that COVID-19 was a new disease, “because the disease was uncertain, the deterioration of disease condition would become very bad, and the patients could not expect the future.” (N16) Secondly, due to the treatment of the disease, the patient required to be isolated from family and friends. Especially if the treatment last for a long time, patients did not understand the policy, and when they saw people around them discharged from hospital, they would have doubts about their own situation. Finally, patients would also worry about recurrence and infection after cured. “Some people say that they are still reluctant to go out even though they have recovered after being discharged from the hospital. They are afraid that going out will increase the burden on everyone and their families. Therefore, everyone’s psychological condition may be different.” (N2) At the same time, negative emotions will accumulate, “if he or she does not find a good way, it will also increase his or her emotional burden.” (N18)

“They will ask us, what should I do if my disease cannot be cured? Will I be locked in this place forever and not allowed to go out? so the patient is still very anxious.” (P3)

According to the working experience of HCWs, “when we observe the patients with a good state of mind, their overall treatment effect and overall recovery will be much better than some anxious patients, including the improvement of mental state and many clinical symptoms, they are much better, much faster.” (P1)

Therefore, patients need particularly psychological attention. “Sometimes patients are not necessarily afraid of pneumonia or virus, after solving the psychological problems, their mood will be relaxed.” (P1)

#### Manifestation of Negative Emotion

Psychological distress must be paid attention to, and how to recognize these manifestations of patients is very critical. “Illness can be treated in accordance with COVID-19 guidelines, but there are no guidelines for psychological problems.” (N2). The patients present different manifestations, including implicit, reverse, explicit, and physiological.

Some patients shown implicit manifestations, such as denying the disease, refusing to communicate with disease-related matters, being silent and alone, passively cooperating with treatment, and feeling guilt, etc.

“Some patients are unwilling to get out of bed. There is a young man in his 20s who is actually not very sick. He would rather play on his mobile phone in bed than communicate with people in the same room.” (N11)

“Some patients said that they could not go out, otherwise they would infect and harm others, and they felt that they were black sheep and criminals. We can understand from his language, in fact, he feels guilty in his heart.” (N15)

Some patients shown a reverse performance. “Although they looked positive, they actually had a lot of thoughts in their hearts, ask about the results of the examination every day and when they can do nucleic acid again. I can feel that he does actually really mind the COVID-19.” (N1)

Although some people will be particularly depressed, reluctant to say, but there were still a number of people mood swings, the performance of overt behavior, such as complaining, crying, distrust of the treatment plan, but also take some inappropriate ways to vent to the doctors and nurses.

“There was a patient called the health institution at night, he felt the hospital had cheated him and locked him up deliberately.” (N15)

“Some patients search a lot of information on the Internet, and will match their own situation with the information they find. In many cases, they cannot understand the current treatment measures, so they show impatience and distrust for HCWs.” (P3)

“Some patients shown physiological manifestations. For example, some patients usually suffer from sleep disorders. They would complain about the harsh conditions of our room and think that the alarm from its equipment disturbed his sleep in a very bad emotional state.” (N3)

#### Manifestation of Proactive Adaptation

Despite the psychological distress, HCWs said that most of the patients were in a relatively good mental health. Under this kind of physical and mental pressure, the patient can still show some positive adaptations. The 362 most obvious of these is that the patient will self-adjust and show an attitude of hope, acceptance, and trust, “I believe the doctor will cure it and overcome this disease,” (N2) “Patients feel they have to be optimistic and accept that in order to overcome the virus.” (P2)

Patients will also make cognitive adjustments, look at the positive side, and learn to be grateful. “Now thanks to the medical policy and good living conditions, they will have the opportunity to receive free concentrated treatment in the isolation ward. If it was in the hard times before, they may be very sick or even dead and have infected many people.” (N10)

“The patients even gained growth and insight. One patient we cared for who was discharged reported that his entire outlook on life had changed and that he was hopeful for the future.” (N1)

In addition to psychological adaptation, some patients also show behavioral adaptation, especially active cooperation with treatment, active exercise, and even take the initiative to help HCWs do some work within their capacity.

“Every day we enter ward, whether we teach patients how to play fitness Qigong, do breathing exercises, or ask about their condition of disease, eating, and sleeping, they are very willing to answer questions one by one.” (N1)

“Sometimes, when we are doing disinfection and sterilization, many patients will take the initiative to help.” (N10)

#### Strategies Relieving Psychological Distress

Healthcare workers attach great importance to psychological care, and perform routine psychological state observation and referral to professional psychological services. Specifically, during ward rounds, doctors in each shift should report the screening work of psychological problems so as to timely conduct psychological intervention, and if not, they will be referred to a professional psychologist. Most patients, as long as they can operate smart phone, they can communicate directly with a counselor through WeChat.

Healthcare workers all stated that the most important thing to deal with patients’ psychological distress is to let patients feel the care, patience and respect from HCWs, so as to build patients’ confidence and trust to HCWs. Most patients’ psychological distress can be alleviated and controlled. “In the past work, most of the missions were about professional knowledge. Here, we communicate with patients more carefully and treat them like their loved ones.” (N4)

It is also crucial to communicate the patients’ disease condition timely and objectively. “HCWs allows patients to know the truth of their disease while inspiring them with examples of curing patients.” (N5) “Let him build confidence, fully understand their treatment plan, fully understand his disease condition, and let him know that we have not given up on him, no one has given up on him, and we are all concerned about him.” (N8)

Secondly, it is also important to meet the patient’s life needs as much as possible, because the patient is in an isolation ward. “As long as he puts forward some reasonable requirements, we will try our best to help him solve the problem and meet his needs. Let him know that when he comes here, we will help him treat the disease thoroughly, so as to gain his trust.” (N5)

Other strategies for alleviating psychological distress include: promoting the emotional connection between patients and HCWs, patients and their families, and patients. “They can also bring their own mobile phones and communicate with their families independently” (N2); as well as listening patiently, they need someone to listen to their thoughts so that they can gradually adjust their mentality.

“We originally planned to buy a cake for a patient for his birthday, but because the epidemic was serious and we couldn’t buy a cake, we brought him a big apple. He was very moved and cried in the ward at that time. He was very grateful to us.” (N13)

“We have a WeChat group, most of the patients will send some positive messages to the group.” (P7)

And some verbal and non-verbal communication skills are also effective, which be used more and more since working in isolation wards. “Even through glass, we will greet patients, communicate through gestures, or provide some household items. I think the more this form of communication, the better for promoting mutual relationship.” (N1)

For verbal communication, we observed and inquired about patients’ physical conditions, or used indirect written expressions.

“……If the patients are embarrassed to say it, I have encouraged them to write on the sticky notes. For example: we are confident to defeat the epidemic with you here, etc.” (N3)

It is also helpful for patients to adjust their emotions and relieve psychological distress by providing them with some ways to divert attention, such as watching TV, doing exercise, listening to music, etc.

#### Gains of Healthcare Workers After Delivering Effective Psychological Care

Although caring for COVID-19 patients can be a challenge, HCWs feel needed and validated after devoting their efforts. “Even if the patient does not say thank you, HCWs will feel very gratified and realized the value of work.” (N1) “Some patients would apologize to me after calming down, explaining that they were out of control and should not have yell at me. I was quite moved.” (N13)

HCWs witnessed a scene of mutual support between patients, which has a great impact on their own values. “Both father and son are sick, father cares about his son especially, father and son depend on each other, support and encourage each other, the scene is still very moving.” (N13)

Patients express their gratitude every day, which empowers and motivates the HCWs, many of whom come from other provinces to support the frontline.

“No matter positive or negative patients, they are very, very grateful to our frontline medical team. Up to now, people in the WeChat group still ask whether we have gone home, thank us and miss the days with us very much.” (N1)

After completing the hard work and successfully evacuating the ward, many HCWs felt the recognition from patients and ordinary residents, which sublimated their sense of professional honor and responsibility, and helped them to be more committed to their career.

“When we left, we 437 saw some residents had written their thanks on old bed sheets and many sanitation workers and police officers waved to us. I was really touched and thought if the epidemic happens again, I would not hesitate to join in the fight.” (N13)

## Discussion

As far as we know, this is one of the few studies that uses a mixed-method design to explore the various factors influencing the psychological distress of COVID-19 patients and the comprehensive views of patients and healthcare workers. These new findings in this study can help fill in the knowledge gaps of COVID-19 patients’ current psychological distress and its influencing factors, and provide the basis for health care workers in areas affected by the epidemic to accurately identify high-risk groups of psychological distress and provide targeted psychological interventions for COVID-19 patients with psychological distress.

### The Current Condition of Psychological Distress and Psychological Care Needs in Patients With COVID-19

In the quantitative survey part, the total score of the psychological distress scale exceeds 4 points, indicating that the patients with COVID-19 have positive psychological distress. In this study, 25.7% of the patients with COVID-19 reported psychological distress, which is lower than other researchers’ finding. Guo et al. conducted a mixed-method study among 103 patients with mild symptoms from 10th to 28th February, 2020 and found that 60.2% patients have depression and 55.3% patients have anxiety. Bo et al. also found that about 96.2% clinically stable patients with COVID-19 in five quarantine facilities have significant posttraumatic stress disorder in March 2020. The reason for the difference may be that this study was implemented in April 2020, which was later than the other two studies. After the acute period of the epidemic, due to the supply of protective materials, the update of diagnosis and treatment plans, the public’s knowledge of epidemic prevention and control has improved, the patients with COVID-19 gradually reduced their fears and worries about the epidemic (Chi, 2020).

However, in the qualitative interviews, healthcare workers also found that there are still some people have psychological problems and particularly need psychological care, which reflect that HCWs should not ignore the psychological distress of patients with COVID-19 when providing care, and psychological care is as necessary as physical care. If these negative emotions are not vented in time, it is easy to cause serious psychological and spiritual problems and affect individual health and social stability. Patients should be encouraged to vent their emotions reasonably and adjust their emotional state, such as writing a diary, communicating with friends and relatives on WeChat and telephone, and calling the psychological counseling hotline, etc. (Gao and Chen, 2003; China Health Education Center, 2020).

### The Manifestation of Psychological Adaptation of Patients With COVID-19

From the perspective of HCWs in qualitative interviews, it is very critical to identify the manifestations of patients’ psychological distress, because the patients with COVID-19 have shown different characteristics, such as implicit, reverse, explicit, and physiological. Therefore, it is necessary for HCWs to recognize these different characteristics and pay close attention to the patients’ psychological condition in order to detect the patients’ psychological distress early and avoid further deterioration of the psychological problems (D’Agostino et al., 2020).

In addition to negative psychological performance, we found that some patients with COVID-19 also show active and positive adaptation to stress, which simultaneously or gradually appeared with negative emotions. Previous 479 studies (Honey and Wang, 2013; Sun et al., 2020) have also shown that patients may gradually adapt to and accept the disease conditions as the disease progresses, they would be confident and hopeful about the treatment, and be adherence to the arrangements of HCWs.

However, the change trajectory of psychological adaptation still needs future research to investigate the COVID-19 patients’ psychological conditions as the patients’ disease progresses. Some studies (Waugh, 2013) have shown that positive emotions play an important role in psychological rehabilitation, which may be also related to the support of patients, family members, government, and social groups in many aspects (Liu and Liehr, 2009; Kang et al., 2018). Therefore, when patients have different types of emotional manifestations, HCWs should provide targeted psychological supports, such as guiding cognitive evaluation, strengthening multi-dimensional social support, and stimulating positive emotions (Bao et al., 2020).

### The Influencing Factors of Psychological Distress of Patients With COVID-19 and Relieving Strategies

Quantitative survey has found that the severity of the patient’s disease is an important factor affecting the patient’s psychological distress. The more severe the patient’s disease, the more significant the psychological distress, which is consistent with previous studies. Li et al. (2021) found that compared with patients who have no significant physical symptoms, patients with physical symptoms are more worried about the progress of the disease. Therefore, for patients with COVID-19 of different severity, it is critical to perform routine psychological state observation and meet the necessary life needs. Furthermore, based on the qualitative interviews, we also found that one of the most effective strategies to relieve psychological distress is to referral patients to professional psychological services. Other studies (Kang et al., 2020) have also found that patients with mild mental disorders prefer to obtain mental health services from the media, while patients with heavier psychological burdens expressed the need to seek services from professionals such as psychologists and psychiatrists. This finding indicates that when screening for psychological problems, it is necessary to take the severity of the disease into consideration, which may be a more direct and specific screening method.

Besides, quantitative survey has also found that patients’ uncertainty about the disease play an important role in affecting patients’ psychological distress, which is similar to the results of other researchers’ studies (Luo et al., 2020). During the outbreak, there are common cognitive biases in all sectors of society, such as prejudice, blind obedience, catastrophe, and coercion, which have caused social panic and rumors to spread. Therefore, reasonable cognitive intervention is particularly important, such as the release of up-to-date, credible and accurate health information, which can help improve public perception, eliminate public panic, and make the general public calmly deal with the epidemic. In addition, the results of qualitative sub-study of this study and other researchers also show that timely disease communication, provision of adequate medical resources, and preventive measures can help reduce patients’ uncertainty about the disease. This highlights the importance of ensuring that preventive measures are taken at the public health system as well as at the individual level to reduce the negative effects of psychological distress.

### Gains of Healthcare Workers After Delivering Effective Psychological Care

At the same time, according to the qualitative interviews, HCWs have achieved professional and spiritual growth after successfully dealing with the psychological distress of patients through multi-disciplinary team cooperation. Although caring for COVID-19 patients is a tough task, HCWs need to face unknown diseases 523 and unpredictable risks, bear heavy workload, high risk of infection, and serious psychological distress (Galehdar et al., 2020), many HCWs still actively join in anti-epidemic fight (Liu Q. et al., 2020). They have involved to help COVID-19 patients avoid suffering pains, especially delivering effective psychological care, thus they would fully fulfill their professional responsibilities, contribute to national security and human wellbeing, and achieve a sense of accomplishment and satisfaction from their work (Wang et al., 2016), in line with previous studies (Kang et al., 2018; Sun et al., 2020). In addition, the HCWs can be continuously appreciated and encouraged by the public and the patients they care for, which boosted their professional pride and sense of identity (Su et al., 2007). Other studies (Fan et al., 2020; Santos, 2020) have also reported that during the epidemic prevention and control period, various epidemic prevention and control knowledge-related trainings provide nurses with opportunities to practice and master skills, which could help them enhance self-efficacy and realize self-worth and gain a sense of professional benefit. Hence, the hospital administrators and educators can actively guide and motivate more HCWs to achieve their own spiritual and professional growth during the epidemic, which may play a positive role in reducing the negative psychological impact of COVID-19 on them.

### Implications for Clinical Practice

The psychological distress of patients with COVID-19 have shown different characteristics, such as implicit, reverse, explicit, and physiological. Therefore, it is necessary for HCWs to recognize these different characteristics and pay close attention to the patients’ psychological condition. Additionally, the severity of the patient’s disease and disease uncertainty are important factors affecting the patient’s psychological distress. With this, it is critical to train the healthcare workers on offering exact and timely information about disease to decrease the uncertainty in illness, and applying communication strategies to relieve psychological distress, which may help the healthcare workers to gain psychological growth and mitigate working stress as well.

## Limitation

When interpreting the current results of this study, several potential limitations need to be considered. First, due to the limited time and energy, the sample size of the quantitative research part of this study was relatively small (only 120 cases), which limited the promotion of the research results to a certain extent. Therefore, it is suggested to expand the sample size in future studies to improve the credibility of the results. Second, considering the risk of infection and limited manpower, researchers cannot go to the sites to collect data face-to-face, and can only conduct convenience sampling in the hospital where the researchers work, which would limit the extrapolation of results. Future studies may, where possible, conduct larger random sampling to improve the reliability of the results. Similarly, due to the urgency of the situation, in order to reduce the risk of infection between interviewers and respondents, data were obtained through video interviews, which would also affect the validity of the results. Besides, although the intrapersonal and interpersonal influencing factors are discussed in this study, the influence factors discussed in this study have limited power to explain the regression equation, indicating that there are more important influencing factors that have not been found, such as the mental health services received, personal personality characteristics, social support, etc. Finally, although this study adopts a mixed-methods design, it is also a cross-sectional level study, as well as an observational study, with the limitations to extrapolating conclusions associated with this kind of study design. In the future, longitudinal research can be carried out to track the change trajectory of psychological distress and provide more evidence for the mechanism of psychological distress in COVID-19 patients.

## Conclusion

In conclusion, the current study has demonstrated that 25.7% of patients with COVID-19 still suffered from psychological distress and showed different characteristics, such as implicit, reverse, explicit, and physiological, which should receive timely attention from healthcare workers. And the severity of the disease and disease uncertainty have a significant impact on distress. It is critical to train the healthcare workers on detecting the different manifestation of inner psychological distress of COVID-19 patients, offering exact and timely information about disease to decrease the uncertainty in illness, and applying communication strategies.

## Data Availability Statement

The raw data supporting the conclusions of this article will be made available by the authors, without undue reservation.

## Ethics Statement

The studies involving human participants were reviewed and approved by the Institutional Review Board at the Researchers’ Institute (IRB number: 202025). The patients/participants provided their written informed consent to participate in this study.

## Author Contributions

AW and XB: concept and design. XB, LL, and XC: data collection. JL and AW: acquisition, analysis, or interpretation of data and drafted the manuscript. All authors discussed the coding throughout the analysis, critically revised the manuscript for important intellectual content, contributed to reviewing and editing the manuscript, and approved the submitted version.

## Conflict of Interest

The authors declare that the research was conducted in the absence of any commercial or financial relationships that could be construed as a potential conflict of interest.

## Publisher’s Note

All claims expressed in this article are solely those of the authors and do not necessarily represent those of their affiliated organizations, or those of the publisher, the editors and the reviewers. Any product that may be evaluated in this article, or claim that may be made by its manufacturer, is not guaranteed or endorsed by the publisher.
